# TP Atlas: integration and dissemination of advances in Targeted Proteins Research Program (TPRP)—structural biology project phase II in Japan

**DOI:** 10.1007/s10969-012-9139-1

**Published:** 2012-05-29

**Authors:** Takao Iwayanagi, Sei Miyamoto, Takeshi Konno, Hisashi Mizutani, Tomohiro Hirai, Yasumasa Shigemoto, Takashi Gojobori, Hideaki Sugawara

**Affiliations:** 1 Center for Information Biology and DNA Data Bank of Japan, National Institute of Genetics, Yata 1111, Mishima, Shizuoka, 411-8540 Japan; 2 Technical Computing Solutions Unit, Makuhari System Laboratory, Fujitsu Limited, Nakase 1-9-3, Chiba, Chiba, 261-8588 Japan; 3 System Department, Tokai Software, Okamiya 1463-86, Numazu, Shizuoka, 410-0011 Japan

**Keywords:** Structural biology, National project, Research dissemination, Targeted Proteins Research Program, Protein 3000 Project

## Abstract

The Targeted Proteins Research Program (TPRP) promoted by the Ministry of Education, Culture, Sports, Science and Technology (MEXT) of Japan is the phase II of structural biology project (2007–2011) following the Protein 3000 Project (2002–2006) in Japan. While the phase I Protein 3000 Project put partial emphasis on the construction and maintenance of pipelines for structural analyses, the TPRP is dedicated to revealing the structures and functions of the targeted proteins that have great importance in both basic research and industrial applications. To pursue this objective, 35 Targeted Proteins (TP) Projects selected in the three areas of fundamental biology, medicine and pharmacology, and food and environment are tightly collaborated with 10 Advanced Technology (AT) Projects in the four fields of protein production, structural analyses, chemical library and screening, and information platform. Here, the outlines and achievements of the 35 TP Projects are summarized in the system named TP Atlas. Progress in the diversified areas is described in the modules of Graphical Summary, General Summary, Tabular Summary, and Structure Gallery of the TP Atlas in the standard and unified format. Advances in TP Projects owing to novel technologies stemmed from AT Projects and collaborative research among TP Projects are illustrated as a hallmark of the Program. The TP Atlas can be accessed at http://net.genes.nig.ac.jp/tpatlas/index_e.html.

**Electronic supplementary material:**

The online version of this article (doi:10.1007/s10969-012-9139-1) contains supplementary material, which is available to authorized users.

## Introduction

Structural biology that seeks to describe the 3-dimensional structures of proteins in correlation with their functions not only serves as the basis for life science research, but also plays a vital role in industrial applications as exemplified in drug developments. Several structural biology projects such as Protein Structure Initiative (http://www.sbkb.org/) and Structural Genomics Consortium (http://www.thesgc.org/about/) are pursuing structures of proteins on a genome wide scale in USA and Europe. In Japan, “National Project on Protein Structural and Functional Analyses” (2002–2006, commonly called “Protein 3000 Project”) funded by the Ministry of Education, Culture, Sports, Science and Technology (MEXT) of Japan contributed to achieve advances in structural biology and to establish the three dedicated centers for structural biology at SPring-8 (http://www.spring8.or.jp/en/), Photon Factory (http://pfwww.kek.jp/index.html), and RIKEN (RIKEN NMR Facility(http://www.ynmr.riken.jp/en/home.html)).

Targeted Proteins Research Program (TPRP, http://www.tanpaku.org/e_index.php) promoted by MEXT, the phase II of structural biology project following the Protein 3000 Project (2002–2006) in Japan, started in 2007 with the five-year plan. By fully utilizing the knowledge and pipelines obtained in the Protein 3000 Project, the Program aims to reveal the structures and functions of the targeted proteins that have great importance in both basic research and industrial applications. To pursue this objective, 35 Targeted Proteins (TP) Projects selected in the three areas of fundamental biology, medicine and pharmacology, and food and environment are tightly collaborated with 10 Advanced Technology (AT) Projects in the four fields of protein production, structural analyses, chemical library and screening, and information platform. Collaboration, especially collaboration between structural analyses and functional analyses, is the key feature of the Program, since both the structural analyses of the selected target proteins guided by the functional information and the functional analyses based on the solved structures have been found to be mutually effective.

In the area of fundamental biology, researchers are embarking on 13 projects to elucidate a variety of biological systems and functions such as proteasome, autophagy and vesicular trafficking through the structural and functional analyses of key proteins involved. In the area of medicine and pharmacology, 10 projects are tackling target proteins and enzymes implicated in diverse diseases from metabolic syndromes to neglected diseases. In the area of food and environment, 12 projects are characterizing important proteins in bacteria, plants, insects, and rodents, which could lead to such beneficial products as antibiotics, modified enzymes and stress-tolerant crops.

Here, the information platform team in the AT Projects summarizes the outlines and achievements of the 35 TP Projects in the system named TP Atlas as part of the dissemination of TPRP. Progress in the diversified areas is described in the modules of Graphical Summary, General Summary, Tabular Summary, and Structure Gallery of the TP Atlas in the standard and unified format. Advances in TP Projects owing to novel technologies stemmed from AT Projects and collaborative research among TP Projects are illustrated as a hallmark of the Program. The TP Atlas can be accessed at http://net.genes.nig.ac.jp/tpatlas/index_e.html.

## Results

### Outline of TP Atlas

**Fig. 1:**
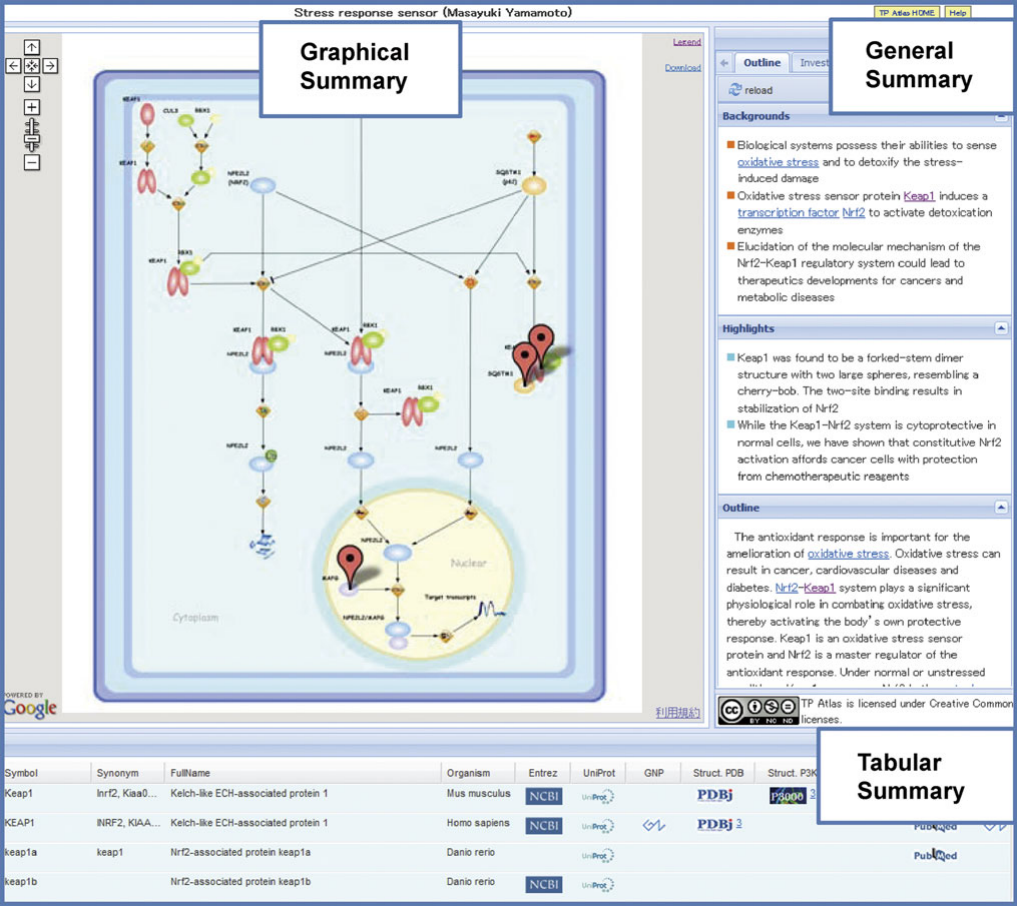
Three modules of TP Atlas. TP (Targeted Proteins) Atlas is a comprehensive “TP Projects achievements database” for all 35 TP Projects. It is composed of the three modules of Graphical Summary, General Summary and Tabular Summary. Given here is TP Atlas of the TP Project: Fundamental Biology B1—Keap1-Nrf2 stress sensor

TP (Targeted Proteins) Atlas (http://net.genes.nig.ac.jp/tpatlas/index_e.html) is a comprehensive “TP Projects achievements database” complied from a variety of information on the target proteins, their structures, published papers and press release for all 35 TP Projects. TP Atlas is composed of the three modules of Graphical Summary, General Summary and Tabular Summary (Fig. 1). It is linked with the TP Structure Gallery which summarizes information on protein structures determined in the TPRP and the P3000 Structure Gallery, a comprehensive collection of structural data produced from the “Protein 3000 Project” (2002–2006) (the preceding project of TPRP). An introductory video for TP Atlas was prepared for those who access the system for the first time.

### Graphical Summary

**Fig. 2:**
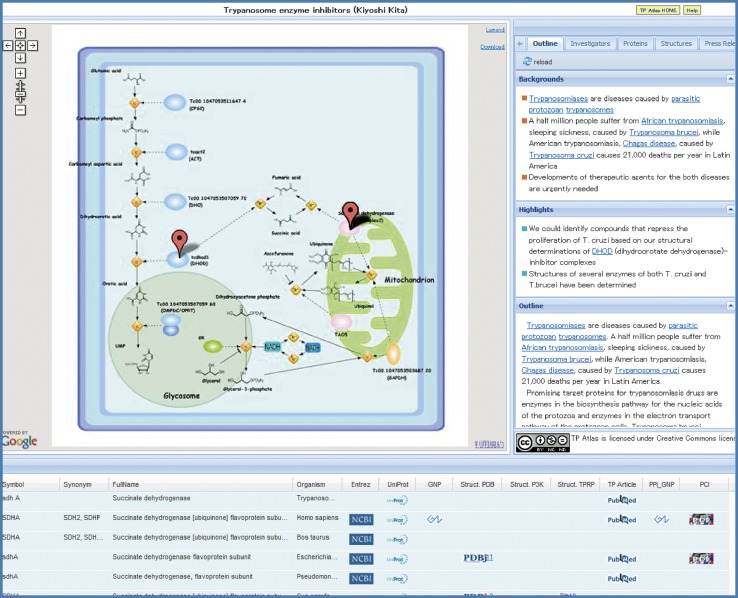
Another example of TP Atlas. Another example of TP Atlas is shown for the TP Project: Medicine and Pharmacology A5—Trypanosome enzyme inhibitors

Signal transduction pathways, protein interaction networks or enzymatic reaction pathways for the target proteins in the 35 TP Projects were depicted in the Graphical Summary in the unified format with Cell Illustrator software (http://www.cellillustrator.com/home). Cell Illustrator is a pathway drawing software developed by Prof. Satoru Miyano and his colleagues at the U. Tokyo [1]. Since it provides with a variety of icons to delineate diversified “unit processes” in cells, we decided to choose Cell Illustrator among many pathway drawing softwares for our application to the TP Projects which deal with various biological processes ranging from signal transduction pathways shown in Fig. 1 to enzymatic reactions pathways drawn in Fig. 2. For each TP Project, the Graphical Summary depicts intracellular and extracellular processes of a pertinent cell as the unit description element in the standard and unified format.

The Graphical Summary for the TP Project of Keap1-Nrf stress sensor [2] was shown in Figure S1, while its entire TP Atlas was shown in Fig. 1 (http://net.genes.nig.ac.jp/networkDB/Ctrl?CI=FBB1&lang=en). The antioxidant response is important for the amelioration of oxidative stress. Nrf2-Keap1 system plays a significant physiological role in combating oxidative stress, thereby activating the body’s own protective response. Keap1 is an oxidative stress sensor protein and Nrf2 (Nfe2l2) is a master regulator of the antioxidant response. Under normal or unstressed conditions, Keap1 suppresses Nrf2 in the cytoplasm. Oxidative stress disrupts the suppression by Keap1, resulting in a build-up of Nrf2. Unbound Nrf2 is then able to translocate into the nucleus, where it will induce many cytoprotective genes including antioxidative enzymes and detoxication enzymes. This series of the cascade was drawn in the Graphical Summary (Figure S1) with 7 distinct molecules and 10 different processes. The meaning of each process icon can be referred by clicking the legend in the upper right of the graph (supplementary material, Figure S2). Each pathway graph can be zoomed in four steps.

**Fig. 3:**
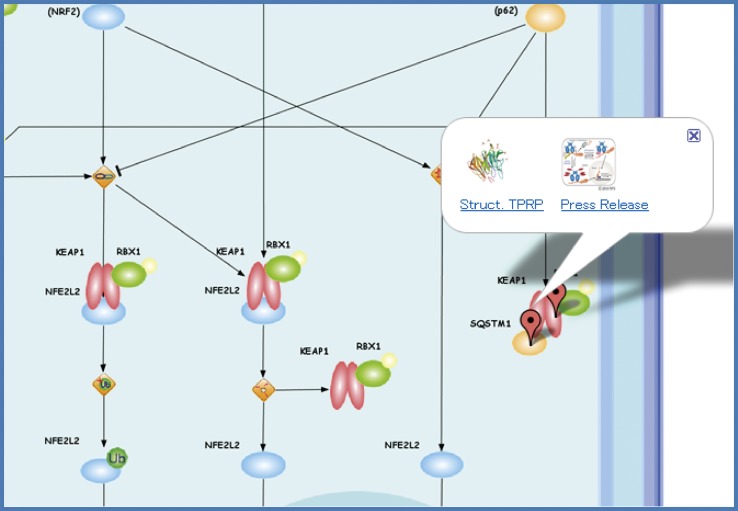
The pop-up balloon for the target protein in the Graphical Summary. The target protein with the solved structure and the corresponding press release in the TP Project was marked with a pop-up balloon to access their detailed information through the corresponding thumb-nailed images

Figure 2 shows another example of TP Atlas for Trypanosomiases (http://net.genes.nig.ac.jp/networkDB/Ctrl?CI=MPA5&lang=en). Two enzymatic reaction pathways, the biosynthesis pathway for the nucleic acids of the protozoa and the electron transport pathway of the protozoan cells, are depicted in the Graphical Summary (see also supplementary material, Figure S3). Trypanosoma brucei possesses an oxidase TAO which is resistant to the poison cyanide and absent in mammalian cells. Ascofuranone produced by mycotic has been found to inhibit TAO [3]. The fourth enzyme DHOD in the biosynthesis pathway for pyrimidine from which nuecleobases are derived is indispensable for protozoan trypanosomes and thereby one of potential targets. Chemical compounds drawn by ChemDraw (http://www.cambridgesoft.com/software/chemdraw/) were imported in the Graphical Summary as images. As shown in Fig. 3, the target protein with the solved structure and the corresponding press release in the TP Project was marked with a pop-up balloon to access their detailed information through the corresponding thumb-nailed images.

As exemplified in the Figs. 1 and 2 (see also supplementary materials, Figures S1 and S3), a variety of intracellular and extracellular biological processes in the all 35 TP Projects ranging from signal transduction pathways to enzymatic reactions pathways were delineated in the unified format with such components as cell, entity and process of Cell Illustrator software. Since the Graphical Summary is described in the format of CSML (Cell System Markup Languages) (http://www.csml.org/), it can be downloaded as a CSML file for further editing and processing with Cell Illustrator.

### General Summary

**Fig. 4:**
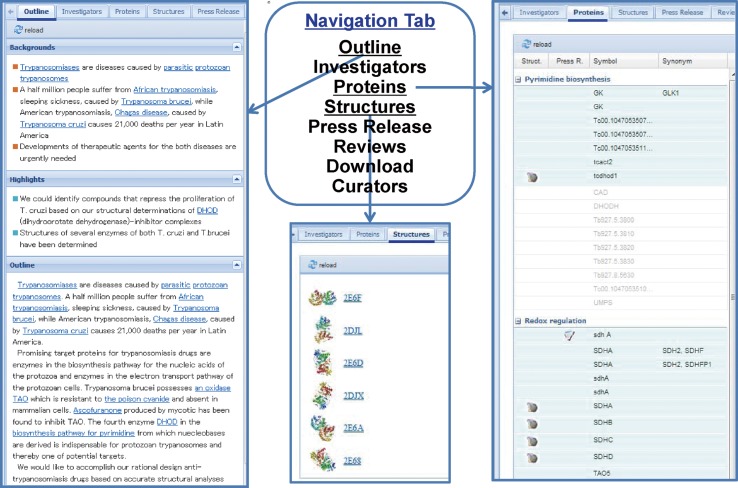
An example of General Summary in TP Atlas. The General Summary is shown for the TP Project: Medicine and Pharmacology A5—Trypanosome enzyme inhibitors. Tab-panel was used to compile and display various information and data in narrow spaces

A variety of information on each TP Project such as the outline (backgrounds, outlines and highlights) of the Project, principal investigator, list of target proteins sorted by subthemes, list of solved structures, press release, published review articles by TPRP participating researchers was summarized in the module of General Summary as shown in Fig. 4. Tab-panel was used to compile and display various information and data in narrow spaces. Data files on TP Structure Gallery, graphs in the Graphical Summary and tables in the Tabular Summary (see below) can be obtained at the download section of the General Summary.

### Tabular Summary

**Fig. 5:**
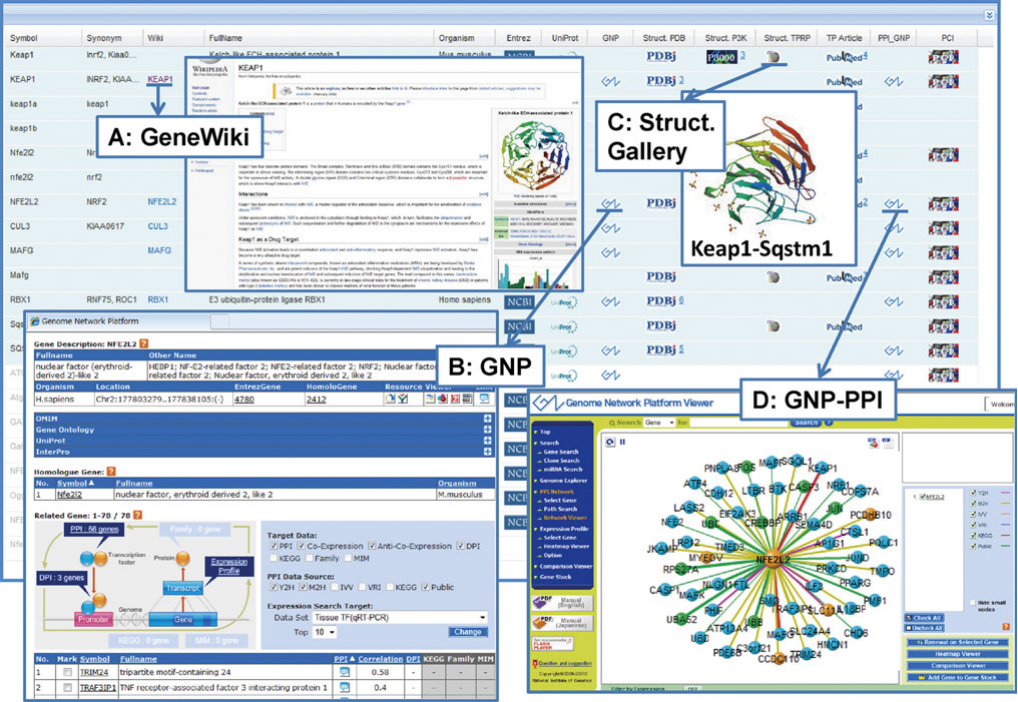
An example of Tabular Summary in TP Atlas. The Tabular Summary is shown for the TP Project: TP Project: Fundamental Biology B1—Keap1-Nrf2 stress sensor

The Tabular Summary for each TP Project is a list of target proteins and their research advances in the form of table. The principal researchers of the TP Projects are obligated to submit annual reports on research proposal, self-assessment, and progress to the MEXT. Information on target proteins and their research progress was gathered from those annual reports as well as research publications collected through the PubMed RSS feed tagged by TPRP participating researchers. An example of the Tabular Summary is given in Fig. 5. Highlighted proteins in the Tabular Summary were included in the Graphical Summary. In addition to Symbol, Synonym, Fullname, Organism, and the links to Entrez Gene and UniProtKB, human proteins were linked to the GeneWiki [4, 5] page in Wikipedia (insert a in Fig. 5) and to the gene page in the Genome Network Project funded by MEXT (http://genomenetwork.nig.ac.jp/index_e.html) (insert b in Fig. 5), which preceded ongoing Cell Innovation Project in Japan (http://www.cell-innovation.org/english/). The structural information for each protein was classified into the three groups of TPRP, P3000 (Protein 3000 Project) (http://mdbpr4.genes.nig.ac.jp/p3k/index.html.en), and other published data and was linked to TP Structure Gallery (see below), P3000 Structure Gallery (see below), and PDBj (http://www.pdbj.org/index.html), respectively (insert c in Fig. 5). Research articles originated from TP Projects were linked to PubMed. Interactor proteins for each human protein can be accessed through the link with protein-protein interaction (PPI) data in the Genome Network Project (insert d in Fig. 5), while binding compounds for each protein can be accessed through the link with protein-compound interaction (PCI) data compiled in PCI-DB (http://chem-web.genes.nig.ac.jp/pci_home_en.html) that collects and merges the four open-access DBs of PubChem (http://www.ncbi.nlm.nih.gov/pcassay), ChEMBL (https://www.ebi.ac.uk/chembl/), DrugBank (http://www.drugbank.ca/), and CTD (Comparative Toxicogenomics Database) (http://ctd.mdibl.org/).

### TP Structural Gallery and P3000 Structure Gallery

**Fig. 6:**
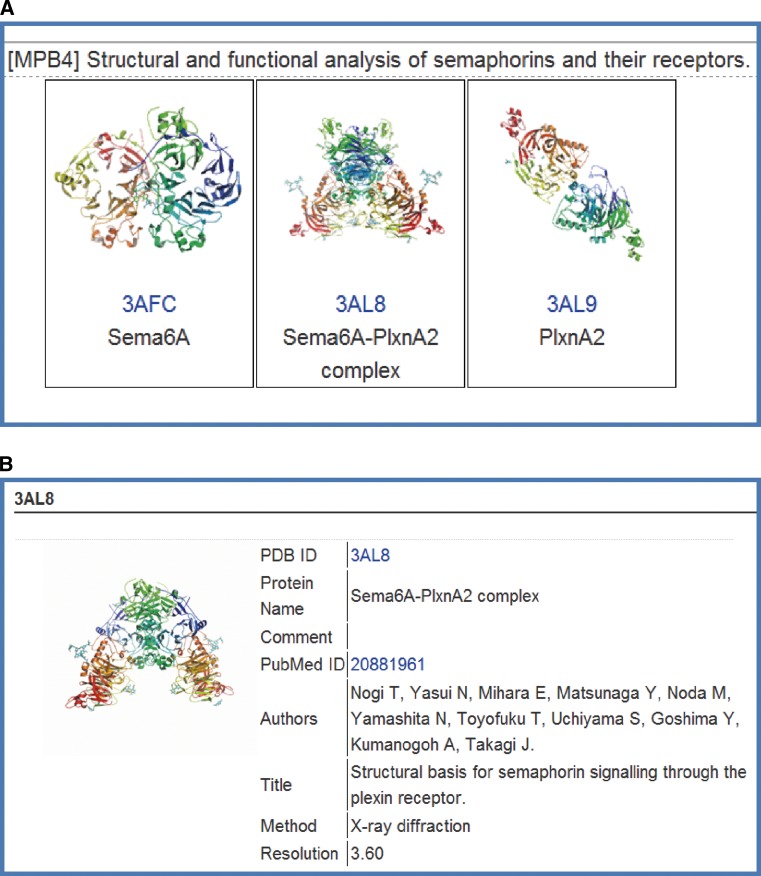
An example of TP Structure Gallery. It consists of the list page of solved structures for each TP Project with structure drawing, PDB code and an abbreviated name of protein (**a**) and the summary page for each structure (**b**)

TP Structure Gallery (http://www.tanpaku.org/tp_gallery/e_index.php) summarizes structural information stemmed from TPRP. It consists of the list page of solved structures for each TP Project with structure drawing, PDB code and an abbreviated name of protein (Fig. 6a) and the summary page for each structure (Fig. 6b). The summary page with the rotating picture of the structure and its corresponding article information linked with PubMed and PDBj can be accessed by clicking the structure drawing in the list page. The whole data of the TP Structure Gallery that include on-hold proteins before PDB data release can be downloaded as an Excel file.

Protein 3000 Structure Gallery (http://mdbpr4.genes.nig.ac.jp/p3k/index.html.en) is a comprehensive collection of structural data produced from “Protein 3000 Project” (2002–2006) (the preceding project of TPRP) funded by MEXT. The Information Platform team of TPRP produces and maintains the site since one of its missions is to disseminate information on TPRP and its related activities.

## Discussion

The outlines and achievements of 35 TP Projects in TPRP were summarized in the system TP Atlas as part of the dissemination of the National Program in Japan. Dissemination is a critical aspect of the mission of large national or international research projects. The post-audit committee of the “Protein 3000 Project” (2002–2006) (the preceding project of TPRP) pointed out the poor sharing and distribution of the Project output. One of the 6 main conclusions of the assessment panel of the Protein Structure Initiative in USA was the poor dissemination of the results (http://www.nigms.nih.gov/News/Reports/PSIAssessmentPanel2007.htm). TP Atlas is a part of efforts in response to the above-mentioned criticism and recommendation. Our emphasis has been put on how the progress in diversified areas could be summarized in the standard and unified format to enable intuitive appreciation for the broad scientific community.

Research advances in the diversified areas are uniformly depicted in the TP Atlas by utilizing both graphical and tabular formats. Figures (Graphical Summary) and plain sentences (Outline) at the entrance of the TP Atlas enable intuitive appreciation of each TP Project without any detailed knowledge of the subject. Cell Illustrator software is particularly suitable for the drawing of the Graphical Summary, since it provides with various icons to delineate diversified “unit processes” in cells. Since graphs in Cell Illustrator are described in the format of CSML (Cell System Markup Languages) (http://www.csml.org/) based on XML, users can download CSML files for further editing and processing.

**Fig. 7:**
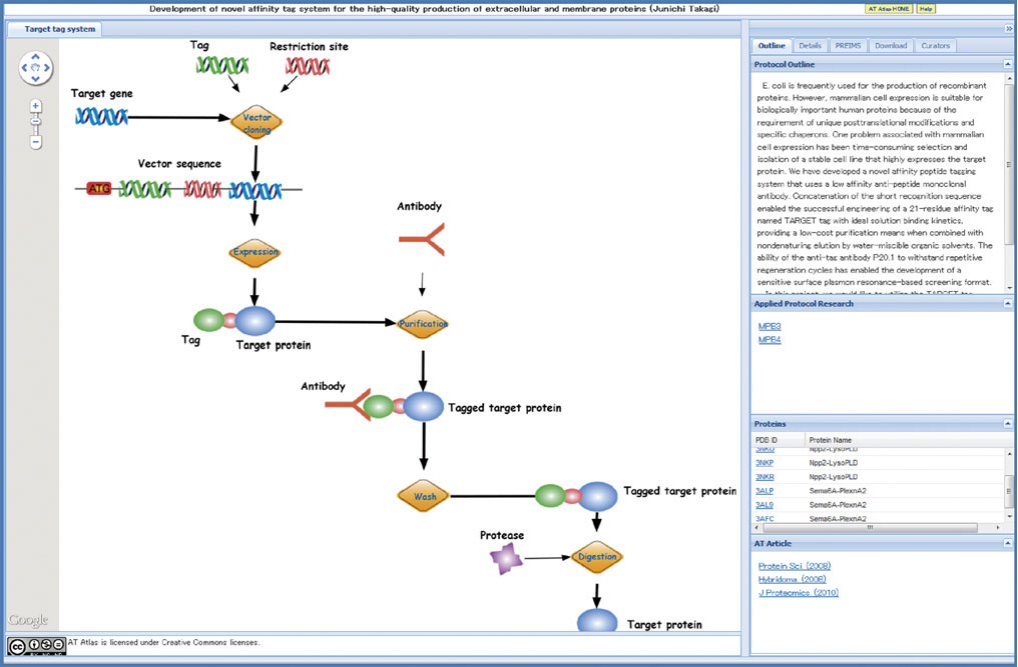
An example of AT Atlas. AT (Advanced Technology) Atlas summarizes progress in 10 AT Projects in TPRP. Given here is AT Atlas of the AT Project: Protein Production D1—TARGET tag

TP Atlas for 35 TP Projects in TPRP is described in this manuscript. AT Atlas that summarizes progress in 10 AT Projects in TPRP is being prepared. An example of AT Atlas is shown in Fig. 7.

Noteworthy advances in AT Projects are beamline developments for future synchrotron radiation protein crystallography including a new micro-beam beamline [6–9] and the construction of public chemical library in Japan for open innovation in drug discovery (http://www.ocdd.u-tokyo.ac.jp/index_e.html). Among more than 350 structures solved in TPRP, worthy of special mention are successful structural analyses of membrane proteins [10–16], protein complex assemblies [17–21], proteins implicated in various diseases [22–24], and plant hormone receptors [25–27].

A typical example of collaborative research among TP Projects is the joint research led by Keiji Tanaka (Fundamental Biology A2: proteasome, TP Atlas: http://net.genes.nig.ac.jp/networkDB/Ctrl?CI=FBA2&lang=en) and Masayuki Yamamoto (Fundamental Biology B1: Keap1-Nrf2 sensor, TP Atlas: http://net.genes.nig.ac.jp/networkDB/Ctrl?CI=FBB1&lang=en). The joint team identified a novel regulatory mechanism by the selective autophagy substrate p62 of the transcription factor Nrf2 through inactivation of Keap1 [28] (see Fig. 3). Advances in TP Projects owing to novel technologies stemmed from AT Projects have been remarkable as a hallmark of the Program. The TARGET tag technology developed by Junichi Takagi et al. [29, 30] in AT Projects (Fig. 7) has been the driving force for the successful structural analyses of two important proteins implicated in various diseases; namely, Semaphorin-Plexin Complex (Medicine and Pharmacology B4: semaphorins & their receptors, TP Atlas: http://net.genes.nig.ac.jp/networkDB/Ctrl?CI=MPB4&lang=en) [22] (see Fig. 6) and Autotaxin (Medicine and Pharmacology B3: ENPP family proteins, TP Atlas: http://net.genes.nig.ac.jp/networkDB/Ctrl?CI=MPB3&lang=en) [23]. The structural information on Autotaxin has paved the way for the development of new inhibitors [31].

## Methods

### System interface

Ext JS library (version 3.0, http://www.sencha.com/products/extjs/) incorporated with AJAX (Asynchronous JavaScript and XML) technology was used to build the TP Atlas system interface. The Ext JS–AJAX combination has enabled the compilation of various information such as Project outline, sorted proteins table, principal investigator, list of solved structures, press releases, and review articles into a page with tab navigation. The usage of panel function in the Ext JS library partitions the overall layout of TP Atlas into 3 parts of Graphical Summary, General Summary, and Tabular Summary.

### Graphical Summary

**Fig. 8:**
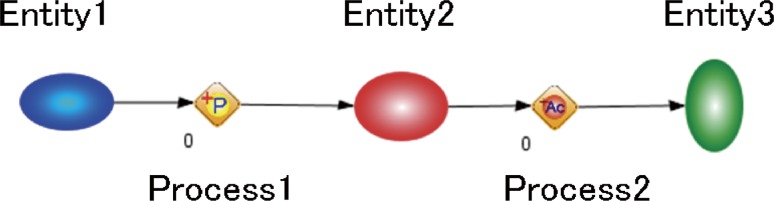
The fundamental diagram of Cell Illustrator. The diagram consists of the two elements of “*entity*” and “*process*”. The entity corresponds to substances such as proteins, RNAs and compounds, while the process indicates unit processes like transcription, modification, binding and cleavage. An “*arrow*” connects an entity with a process alternately. The legend of the processes is shown in Figure S2

Cell Illustrator software (http://www.cellillustrator.com/home) [1] developed for drawing of biological processes based on Petri net was used to create the Graphical Summary of TP Atlas. Its GUI design is easy to grasp intuitively compared with various network analysis and visualization tools such as Cytoscape (http://www.cytoscape.org/) and CellDesigner (http://www.celldesigner.org/). Furthermore, it provides with a variety of icons to delineate diversified “unit processes” in cells. Therefore, we decided to choose Cell Illustrator for our application to the TP Projects which deal with various biological processes ranging from signal transduction pathways shown in Fig. 1 to enzymatic reactions pathways drawn in Fig. 2. The fundamental diagram of Cell Illustrator consists of the two elements of “entity” and “process” as shown in Fig. 8. The entity corresponds to substances such as proteins, RNAs and compounds, while the process indicates unit processes like transcription, modification, binding and cleavage. An “arrow” connects an entity with a process alternately. The connection between entities or between processes is inhibited. There are three types of arrows; an arrow that indicates process sequence, an arrow that represents enzymatic involvement, and an arrow that displays reaction inhibition (see Fig. 2). Although more than 200 icons are available for both the entities and the processes, some of icons in the Graphical Summary were drawn separately. “Cell component” icons that depict cells, cell membranes and various organelles are also available for background images. Majority of background images in the Graphical Summary was the cell graph with some exceptions of mitochondrion and cell membranes.

## Electronic supplementary material

Below is the link to the electronic supplementary material.

Supplementary material 1 (PDF 66 kb). An example of Graphical Summary in TP Atlas. The Graphical Summary is shown for the TP Project: Fundamental Biology B1—Keap1-Nrf2 stress sensor with 7 distinct molecules and 10 different processes

Supplementary material 2 (PDF 133 kb) Legend for biological processes in Cell Illustrator. The meaning of each process icon of Graphical Summary can be referred by clicking the legend in the upper right of the graph

Supplementary material 3 (PDF 58 kb) Another example of Graphical Summary in TP Atlas. The Graphical Summary is shown for the TP Project: Medicine and Pharmacology A5—Trypanosome enzyme inhibitors
