# Supplementary figures and images for: TP Atlas: integration and dissemination of advances in Targeted Proteins Research Program (TPRP)—structural biology project phase II in Japan
Source: J Struct Funct Genomics. 2012 May 29;13(3):145–54. doi: 10.1007/s10969-012-9139-1 (PMC3414706; doi:10.1007/s10969-012-9139-1)

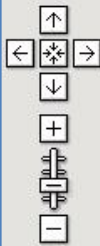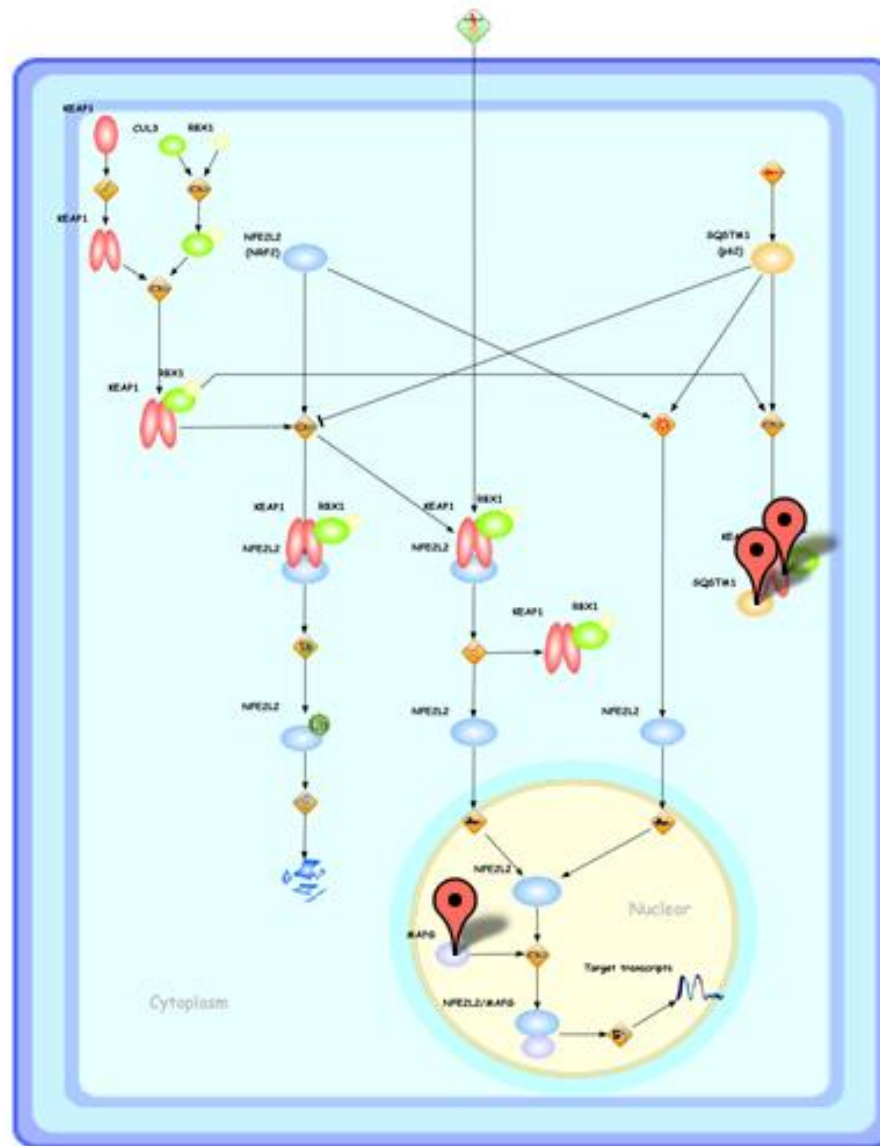

[Legend](#)

[Download](#)

Supplement: Supplementary file 1 — Supplementary material 1 (PDF 66 kb). An example of Graphical Summary in TP Atlas. The Graphical Summary is shown for the TP Project: Fundamental Biology B1—Keap1-Nrf2 stress sensor with 7 distinct molecules and 10 different processes [file 10969_2012_9139_MOESM1_ESM.pdf]
