# Supplementary material for: TP Atlas: integration and dissemination of advances in Targeted Proteins Research Program (TPRP)—structural biology project phase II in Japan
Source: J Struct Funct Genomics. 2012 May 29;13(3):145–54. doi: 10.1007/s10969-012-9139-1 (PMC3414706; doi:10.1007/s10969-012-9139-1)
Supplement: Supplementary file 2 — Supplementary material 2 (PDF 133 kb) Legend for biological processes in Cell Illustrator. The meaning of each process icon of Graphical Summary can be referred by clicking the legend in the upper right of the graph [file 10969_2012_9139_MOESM2_ESM.pdf]

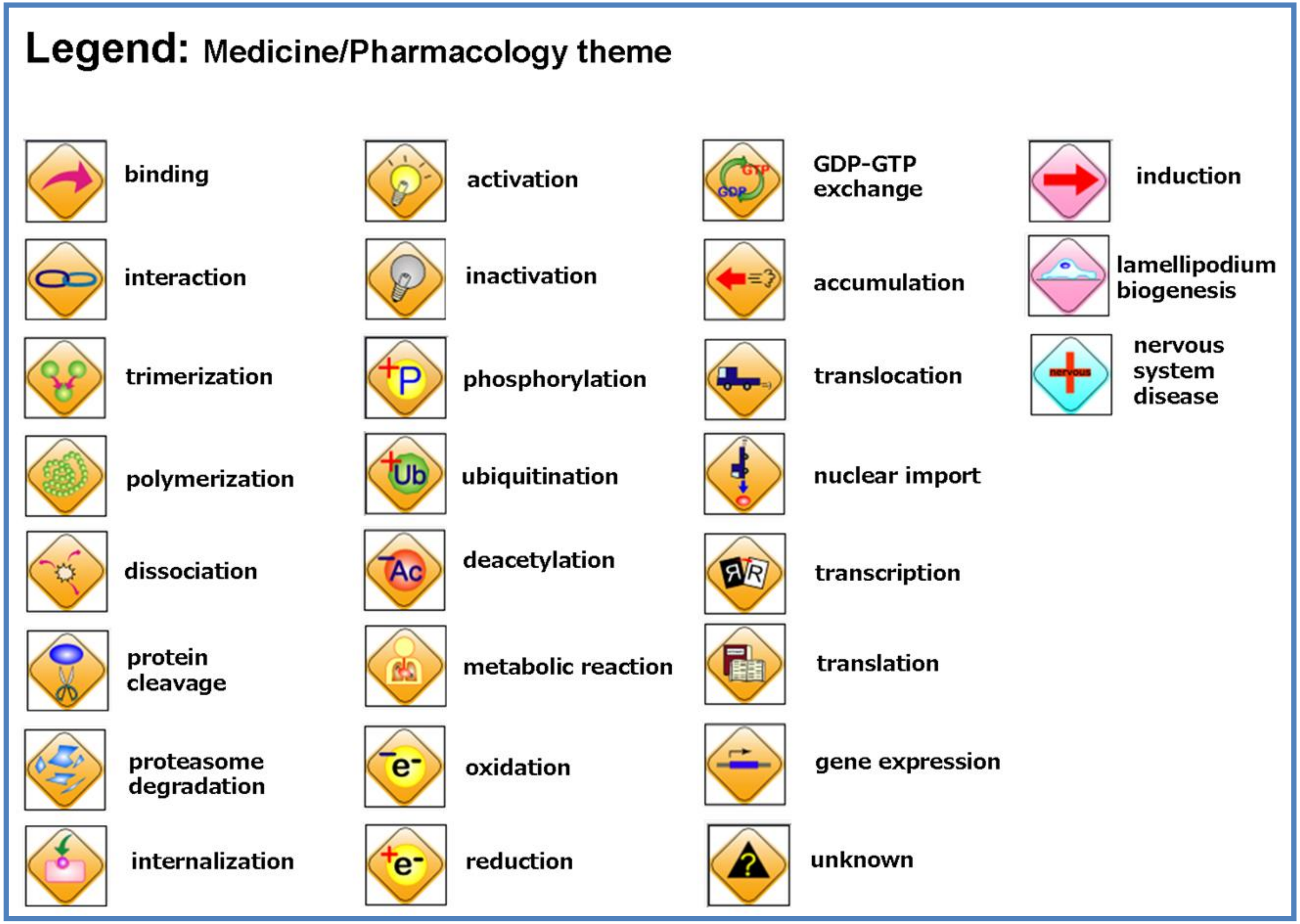
Legend: Medicine/Pharmacology theme
binding
interaction
trimerization
polymerization
dissociation
protein cleavage
proteasome degradation
internalization
activation
inactivation
phosphorylation
ubiquitination
deacetylation
metabolic reaction
oxidation
reduction
GDP-GTP exchange
accumulation
translocation
nuclear import
transcription
translation
gene expression
unknown
induction
lamellipodium biogenesis
nervous system disease
