# Supplementary material for: TP Atlas: integration and dissemination of advances in Targeted Proteins Research Program (TPRP)—structural biology project phase II in Japan
Source: J Struct Funct Genomics. 2012 May 29;13(3):145–54. doi: 10.1007/s10969-012-9139-1 (PMC3414706; doi:10.1007/s10969-012-9139-1)
Supplement: Supplementary file 3 — Supplementary material 3 (PDF 58 kb) Another example of Graphical Summary in TP Atlas. The Graphical Summary is shown for the TP Project: Medicine and Pharmacology A5—Trypanosome enzyme inhibitors [file 10969_2012_9139_MOESM3_ESM.pdf]

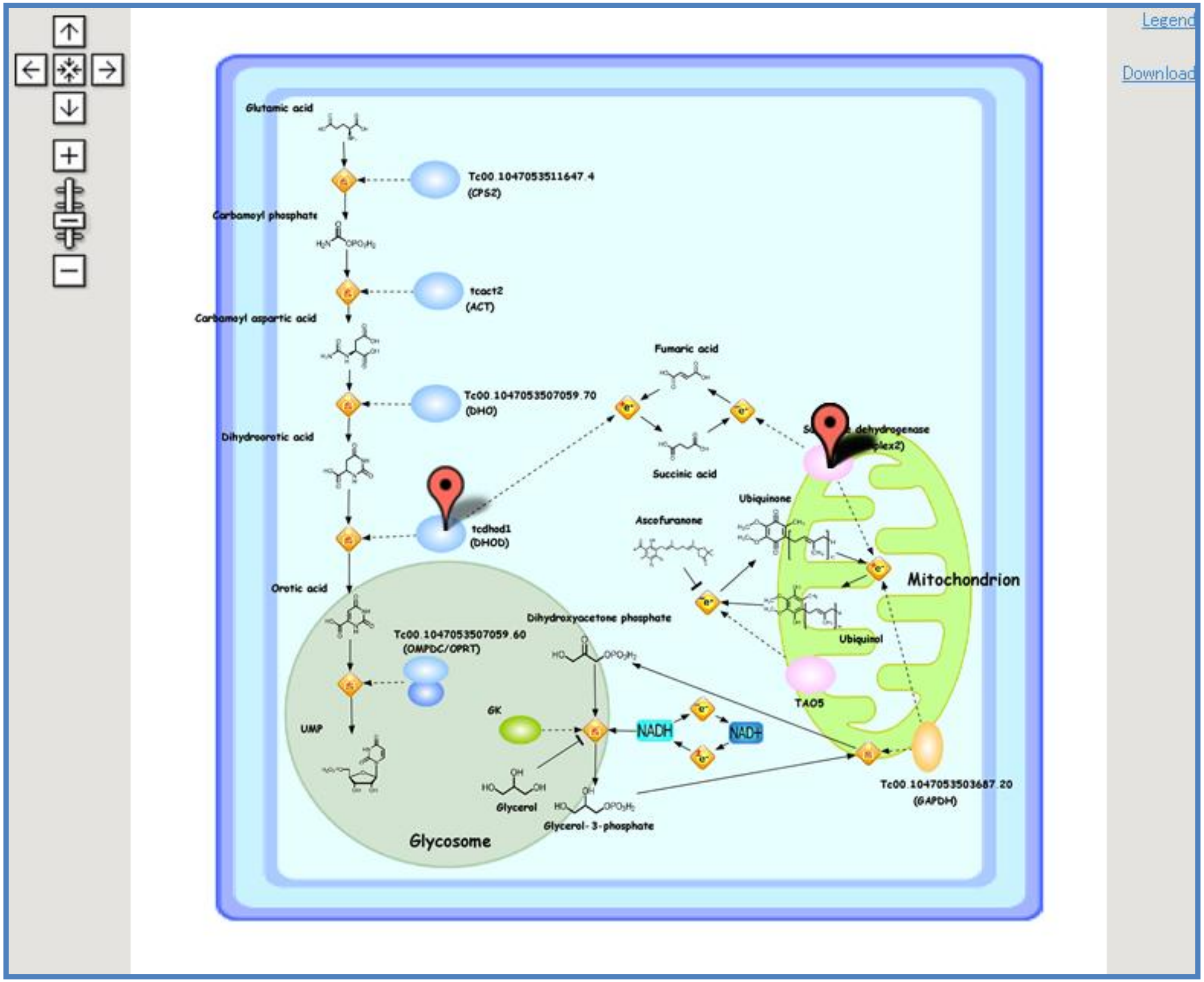
Legend
Download
Glutamic acid
Tc00.1047053511647.4
(CPS2)
Carbamoyl phosphate
tcact2
(ACT)
Carbamoyl aspartic acid
Tc00.1047053507059.70
(DHO)
Dihydroorotic acid
tcdhod1
(DHOD)
Orotic acid
Tc00.1047053507059.60
(OMPDC/OPRT)
UMP
Fumaric acid
Succinic acid
Ubiquinone
Ascofuranone
Ubiquinol
Mitochondrion
TAO5
Dihydroxyacetone phosphate
GK
NADH
NAD+
Glycerol
Glycerol-3-phosphate
Glycosome
Tc00.1047053503687.20
(GAPDH)
